# Setting Mechanism of a CDHA Forming α-TCP Cement Modified with Sodium Phytate for Improved Injectability

**DOI:** 10.3390/ma12132098

**Published:** 2019-06-29

**Authors:** Jan Weichhold, Uwe Gbureck, Friedlinde Goetz-Neunhoeffer, Katrin Hurle

**Affiliations:** 1 Department for Functional Materials in Medicine and Dentistry, University of Wuerzburg, Pleicherwall 2, 97070 Würzburg, Germany; 2 GeoZentrum Nordbayern-Mineralogy, Friedrich-Alexander-University of Erlangen-Nuernberg, Schlossgarten 5a, 91054 Erlangen, Germany

**Keywords:** calcium phosphate cement, phytic acid, in-situ XRD, injectability, rheology

## Abstract

A calcium deficient hydroxyapatite (CDHA) forming cement with a bimodal grain size distribution, composed of α-TCP and fine grained CDHA at a weight ratio of 9:1, was modified by the addition of sodium phytate (IP6) in variable amounts ranging from 0.25 to 2 wt.%, related to the powder content. The injectability of the cement paste was drastically increased by the IP6 addition, independent of the amount of added IP6. Additionally, the cement paste viscosity during the first minutes decreased. These effects could be clearly related to a slightly more negative zeta potential. Furthermore, IP6 was shown to strongly retard the setting reaction, as can be seen both in the calorimetry and X-ray diffraction measurements. In addition, octacalcium phosphate (OCP) was identified as a further setting product. All measurements were performed at 23 °C and 37 °C to assess the effect of temperature on the setting reaction for both clinical handling by the surgeon and the final hardening in the bone defect.

## 1. Introduction

Calcium phosphate cements (CPCs) are well established in medicine for bone repair and a variety of formulations are commercially available for clinical applications [1]. Compared to calcium phosphate ceramics, they offer the advantage of being freely moldable and therefore perfectly adjustable to the bone defect. CPCs can be basically divided into apatite and brushite cements, according to their final hydration product. While brushite forms under acidic conditions at a pH < 4.2 and a physiological temperature, apatite formation occurs at neutral and alkaline pH values [2]. Apatite and brushite cements differ in their application-relevant properties. Hence, both types are of clinical interest, dependent on the properties required for specific applications. While brushite cement stands out by its high resorbability, apatite cement is advantageous with respect to its mechanical performance [3]. One approach to generate an apatite forming cement is the mixture of α–tricalcium phosphate (α–TCP) with water, which forms calcium-deficient hydroxyapatite (CDHA) according to Equation (1) [4]:

3 α-Ca_3_(PO_4_)_2_ + H_2_O → Ca_9_(PO_4_)_5_(HPO_4_)OH (1)

For a successful clinical application of bone cements, several aspects must be considered. One important factor is the setting time, which needs to be slow enough to give the surgeon enough time for implementation, but fast enough not to delay the operation [5]. Since the hydration of crystalline α–TCP with water alone is rather slow, acceleration is necessary. One possibility is the application of Na_2_HPO_4_, which takes advantage of the common ion effect [6]. Furthermore, the addition of nanosized CDHA as a nucleation agent can fasten the reaction [7]. 

Due to the increasing relevance of minimal-invasive applications, where the cement is directly applied to the defect by a syringe, the injectability of the cement pastes is another highly relevant aspect [8]. Different approaches were already developed to alter cement rheology and to improve injectability [9]. Although increasing the liquid to powder (L/P) ratio has a positive effect [10], it has the drawback that the mechanical properties are negatively affected due to an increase of cement porosity [1]. Second, the particle size of the cement powder can be adjusted to optimize injectability. While a certain decrease of particle size was shown to improve injectability, a prolonged milling leads to a reduction of injectability due to particle agglomeration, accompanied by a decrease in compressive strength [11]. A more efficient improvement can be achieved by adjusting a bimodal grain size distribution, for example through the addition of fine-grained filler particles [12]. Since the setting reaction occurring in cements negatively affects the injectability, the application of setting retarders can also be beneficial [13]. Another approach is the alteration of the zeta potential of cement particles by the addition of multiple charged ions. The resulting repulsion between particles of the same charge leads to a decrease of viscosity and an improved injectability [14]. This effect was already described for citric acid and sodium citrate, respectively, in both brushite and apatite forming cements [14,15]. Unfortunately, the application of citric acid can also have some drawbacks. For instance, previous studies indicated that the addition of citric acid was detrimental for cell attachment and the degradation of the cement *in vivo* [16,17]. Therefore, the development of alternatives is of interest. A rather new approach is the application of phytic acid (IP6), which was already demonstrated to be cytocompatible and effective as a setting retarder in brushite cement [18]. While the effect of IP6 on cement setting, combined with a detailed study of cement paste rheology, was already analyzed in detail for a brushite cement [19], according to our knowledge no apatite cements modified with IP6 derivates were investigated so far. 

Hence, the aim of the present study was the development of an injectable CDHA-forming cement based on α–TCP, which might open up new fields for application. Fine grained CDHA was added as a filler to adjust a bimodal grain size distribution. As the addition of phytic acid might result in a pH too low for CDHA formation, sodium phytate was added instead in variable amounts to investigate its effect on cement injectability and rheology. Furthermore, the influence of the additive on cement hydration at two different temperatures was investigated in detail using isothermal calorimetry and quantitative X-ray diffraction (XRD), since previous studies indicated a general acceleration of the hydration reaction with an increasing temperature through the application of isothermal calorimetry [20,21,22]. A detailed understanding of the hydration process is an essential prerequisite for the targeted development of bone cements for clinical applications. 

## 2. Materials and Methods 

### 2.1. Powder Fabrication and Characterization 

#### 2.1.1. Powder Fabrication 

For the synthesis of α–TCP, monetite (CaHPO_4_, Honeywell, USA) and calcite (CaCO_3_, Merck, Germany) were mixed in a molar ratio of 2:1 in a planetary ball mill (PM 400, Retsch) for 1 h. The powder mixture was sintered at 1400 °C for 5 h. Afterwards, the sintered powder was finely ground in the planetary ball mill for 1 h.

The apatite was synthesized by stirring α–TCP suspended in 1 l deionised water with the addition of 30 ml 2.5% Na_2_HPO_4_ solution. Due to the synthesis procedure, it can be assumed that it is a calcium-deficient hydroxyapatite (Ca_(10−x)_(HPO_4_)_x_(PO_4_)_(6−x)_(OH)_(2−x)_, CDHA) (see Equation (1)). After 7 d, the suspension was filtered and the received powder was dried at 60 °C in an oven. Both powders were analysed for phase purity via an XRD analysis. In order to obtain a fine powder, the dried CDHA precipitate was wet milled in a planetary ball mill for 2 h in agate grinding jars. For 10 g of CDHA powder, 175 g of ZrO_2_:Y beads with a diameter of 1.25 mm were used as the grinding medium and 10 ml of isopropyl alcohol as lubricant. After milling, pure CDHA powder was obtained by separating the ZrO_2_:Y beads with a metal sieve and centrifuging the residual slurry at 3500 rpm for 90 min in a centrifuge Megafuge 1.0 (Heraeus, Hanau, Germany). Then, the supernatant isopropyl alcohol was removed with a pipette, and the samples were dried at 50 °C in a vacuum drying chamber (Binder, Tuttlingen, Germany) to remove the residual ethanol.

#### 2.1.2. Particle Size Distribution and Specific Surface Area

The particle size distributions of the α–TCP and CDHA starting powders were determined by laser diffraction at a Mastersizer 3000 (Malvern Panalytical, Kassel, Germany), applying the Mie scattering model. Isopropyl alcohol was used as a dispersing agent. A data evaluation was performed using Mastersizer software V3.5 and Microcal Origin V 2017G. Three independent preparations of each sample were measured, and 10 measurement runs of each preparation were performed. The BET (Brunauer, Emmett, Teller) surface area of α–TCP and CDHA was determined by Nitrogen adsorption using a Gemini 2360 instrument (Micromeritics, Norcross, GA, USA) in liquid N_2_. The samples were prepared for measurements by cleaning the surfaces at 130 °C under He flow for 3 h. Three independent measurements were performed for each powder.

#### 2.1.3. Quantitative Phase Composition 

The quantitative phase composition of the powders was determined by powder X-ray diffraction (XRD) combined with Rietveld refinement and the G-factor method, an external standard method [23]. A quartzite slice, calibrated with fully crystalline silicon powder (NIST Si Standard 640d), was used as an external standard. The details about the application of the G-factor method for the investigation of α–TCP cements can be found in [24]. XRD measurements were performed at a D8 Advance with DaVinci design (Bruker AXS, Karlsruhe, Germany) with the following measurement parameters: Range 9–70° 2θ; step size 0.0112° 2θ, integration time 0.2 s; radiation: copper K_α_; generator settings: 40 kV, 40 mA; divergence slit: 0.3°; sample rotation with 30 min^−1^.

For the Rietveld refinement, the structures ICSD# 923 (α–TCP) [25], ICSD# 97500 (β–TCP) [26] and ICSD# 26204 (hydroxyapatite, used for the refinement of CDHA) [27] were applied together with a Chebychev polynomial of second order for the background. The refined parameters were scale factors, lattice parameters, crystallite size and microstrain. The anisotropic crystallite (coherent scattering domain) sizes of the hexagonal CDHA were modelled by a triaxial ellipsoid model [28]. Due to the constraints of the hexagonal symmetry, rx and ry were set to the same value. rx was aligned parallel to the direction of the crystallographic a-axis and rz to the direction of the crystallographic c-axis. The “true crystallite size” (True CS) was calculated as the cube root of the model ellipsoid volume. The contribution of the amorphous TCP (ATCP) present in the samples was modelled with a Peaks Phase, which means that several separate peaks were added to model the contribution of the X-ray amorphous phase. 

#### 2.1.4. Zeta Potential

The effect of sodium phytate on the zeta potential ζ of the particles in the cement paste was investigated with the Zetasizer Nano (Malvern, Germany). For each measurement, 1 g α–TCP, CDHA or a mixture of α–TCP/CDHA in the ratio 9:1 was suspended either in a 0.002 M sodium phytate solution or as a reference in deionized water, which served as a medium for the measurement. Each mixture was prepared 3 times, while each preparation resulted in 3 measurements.

### 2.2. Cement Paste Composition and Characterization 

#### 2.2.1. Paste Composition

For all tests aside from the zeta potential measurement, the powder mixture (P) consisted of 90 wt.% α–TCP, 10 wt.% CDHA and either no sodium phytate or 0.25, 0.5, 0.75 or 1.0 wt.% sodium phytate added related to the mass of the phosphate powder for the samples Phy_0.00, Phy_0.25, Phy_0.50, Phy_0.75 and Phy_1.00 , respectively. Additionally, samples with 2 wt.% sodium phytate (Phy_2.00) were prepared for XRD measurements after hardening, in order to systematically investigate the effect of phytate on OCP formation. Due to the very slow hardening of this paste, it was not included in the other investigations. The liquid phase (L) for the zeta potential measurements was a 0.05 M sodium phytate solution, but for every other measurement a 0.2 M Na_2_HPO_4_ aqueous solution was used. A liquid to powder ratio (L/P) of 0.3 ml/g was used for all experiments except the zeta potential and pH measurements. 

#### 2.2.2. Viscosity

The development of the viscosity η of Phy_0.25 and Phy_1.00, to compare the effect of relatively low and relatively high amounts of added sodium phytate, was documented using an MCR 310 Rheometer (Anton Paar, Ostfildern-Scharnhausen, Germany) at room temperature and at 37 °C. For all the measurements, the measuring unit consisted of two equally sized plates with the upper plate having a circular area with a diameter of 50 mm. The lower plate was fixed, while the other plate was rotating in a defined distance of 1.0 mm above it. For the viscosity measurement, the upper plate rotated with a constant shear rate *γ = 11/s*. The measurements were reproduced 3 times for each composition. A representative measurement was chosen for comparison.

#### 2.2.3. Injectability

The injectability of the different pastes, consisting of α–TCP, CDHA and the respective amount of sodium phytate mixed with 0.2 M aqueous NaHPO_4_ solution (L/P = 0.3 ml/g), was measured using a custom syringe mount and the universal testing machine Z010 by Zwick. A 5 ml syringe equipped with a 2 mm diameter needle was used. 2, 5 and 10 min after mixing of the solid and liquid, the syringe filled with the cement paste was mounted in the Z010 universal testing machine, and pressure was applied at a cross-head speed of 30 mm/min. The measurement was finished either after the entire paste was pressed out of the syringe or the maximum force of 300 N was reached. Afterwards, the injectability was calculated as the percentage difference between the initially loaded paste and the remaining amount at the end of the measurement.

### 2.3. Hydration Reaction

#### 2.3.1. Heat Flow Calorimetry

Isothermal heat flow calorimetry was conducted at a TAM Air isothermal calorimeter (TA Instruments, New Castle, DE, USA) equipped with eight twin type channels consisting of a sample and a reference chamber. It has an integrated thermostat with a temperature variance of ± 0.02 °C. The temperature of the calorimeter was adjusted at 23 °C and 37 °C, respectively. Internal stirring using InMixEr (Injection & mixing device for internal paste preparation, FAU Erlangen, Mineralogy) was applied, while stirring was performed for 1 min using an external motor with a constant stirring rate of 858 rpm. This method already provides reliable data for the initial part of the reaction, since disturbances by the opening of the measurement channels can be avoided. The cement mixtures containing sodium phytate were homogenized in a mixer mill Retsch MM 200 (Retsch, Haan, Germany) in a Teflon tool with four steel grinding balls for 2 × 2 min to ensure the homogeneous distribution of the phytate in the sample. The homogenized powder mixtures were then inserted into the calorimeter crucibles for the measurement. The equilibration of the samples before measurement was performed directly in the measurement channels. Three independent measurements of each sample were performed. The evaluation of the measurements was performed by the software Microcal Origin V 2017G. The heat flow curves were corrected for the calibration constant of the InMixEr tools and the time constant [29]. The calorimetry curves were integrated to obtain the total heat release during hydration.

#### 2.3.2. In-situ XRD

Quantitative in-situ XRD measurements of α–TCP, Phy_0.00, Phy_0.25 and Phy_0.50 at 37 °C and of Phy_0.00, Phy_0.25 and Phy_0.50 at 23 °C were performed at a D8 Advance with a DaVinci design diffractometer (Bruker AXS, Karlsruhe, Germany). Cements with higher phytate concentrations were not measured due to the slow setting of these pastes. The following measurement parameters were applied: Range 3–50° 2θ; step size 0.0112° 2θ, integration time 0.2 s; radiation: copper K_α_; generator settings: 40 kV, 40 mA; divergence slit: 0.3°. One range was recorded every 15 min. The desired temperature was adjusted by a Peltier element. The powder and liquid were equilibrated at 37 °C in a drying oven (Memmert, Schwabach, Germany) and in an Erlanger calorimeter at 23 °C, respectively, for at least 3 h. The cement pastes were then prepared by mixing liquid and powder for 1 min with a metal spatula, filled into a special sample holder and covered with a Kapton polyimide film (Chemplex Industries, Cat. No. 440, Palm City, FL, USA) to minimize water evaporation. Three independent measurements were performed for each sample. The overall measurement time was 24 h at 37 °C and 48 h at 23 °C. 

Each single range was evaluated by Rietveld refinement combined with the G-Factor method to obtain the quantitative development of the phase composition. The lattice parameters of α–TCP starting powder were fixed to the data obtained from the powder refinement. In addition to the structures used for the powder refinement, the structure ICSD #65347 [30] was applied for the refinement of octacalcium phosphate (OCP). The background contributions of water and the Kapton film were both modelled by a hkl phase [31]. For 37 °C, the data were corrected for the water loss occurring during the in-situ XRD analysis. For this purpose, the amount of residual water present at the end was determined by weighing the sample before and after drying at 60 °C in a drying oven (Memmert, Schwabach, Germany). A linear water loss during the measurement was assumed.

#### 2.3.3. pH Development

The pH measurements were performed with an InoLab Level 1 pH meter, equipped with a SenTix 61 pH electrode (Xylem Analytics Germany Sales GmbH & Co. KG, WTW, Weilheim, Germany). The electrode was filled with 3 M KCl electrolyte. The pH of the 0.2 M Na_2_HPO_4_ mixing liquid was recorded, as well as the pHs of the mixing liquids containing the same amount of sodium phytate as the cement pastes used for the other investigations. For this purpose, the corresponding amount of sodium phytate was dissolved into the 0.2 M Na_2_HPO_4_ aqueous solution. Furthermore, the pH of Phy_0.00 and Phy_0.25 was recorded over time to investigate the effect of the sodium phytate addition on the pH development during hydration. The P/L of the cement pastes was increased to 1.2 ml/g to ensure that the cements remained paste-like during the process of hydration and that the pH electrode could be removed afterwards. Hence, the actual content of sodium phytate in the solution decreased to 1/4 and would correspond to an amount of 0.0625 wt.%. The pastes were filled into centrifuge tubes after mixing for 1 min with a metal spatula. The tubes were then stored in a water bath adjusted at 37 °C for hydration. After certain time intervals, the cement pastes were loosened with a metal spatula, and the pH electrode was inserted for measurement. For both compositions, three independently prepared measurements were performed to check reproducibility. The measurements were performed over 9 h to cover the main part of the reaction. 

### 2.4. Characterization of Hydration Products 

#### 2.4.1. XRD of Hardened Samples

In order to investigate the quantitative phase composition after different time points, the mixed cement pastes were prepared into special plastic containers (inner diameter 23 mm; inner height 3 mm) that could be tightly closed with a lid. The closed containers were further sealed with Parafilm. By this means, water loss during the storage of the samples was avoided. In addition to the pastes Phy_0.00 – Phy_1.00, a sample with 2 wt.% sodium phytate (Phy_2.00) was also measured here to further investigate the OCP formation. The samples were allowed to harden in a drying oven (Memmert, Schwabach, Germany) at 37 °C for 1 d or 7 d, respectively, or in an Erlanger calorimeter at 23 °C for 1 d, 2 d, 4 d or 7 d. After hardening, the lid was removed and the sample surface was polished with grit 180 grinding paper. The samples were then prepared into special sample holders and covered with Kapton polyimide film (Chemplex Industries, Cat. No. 440, Palm City, FL, USA) for XRD measurements; an angle range of 3° to 70° (2θ) was measured, and the other parameters were the same as for in-situ XRD measurements. Three independently prepared samples were analyzed for each temperature, time and sample composition. The quantitative phase composition was then determined by Rietveld refinement and G-factor quantification, applying the same procedure as for in-situ XRD. 

#### 2.4.2. Compressive Strength

The samples for the compressive strength (CS) tests were prepared as follows: each sample batch consisted of 14.4 g α–TCP, 1.6 g CDHA and the respective amount of Na–phytate ranging from 0 to 1 wt.% related to the total amount of cement powder. Every powder was mixed with 4.8 ml of an 0.2 M NaHPO_4_ aqueous solution (L/P = 0.3 ml/g) and was stirred for 1 min. Then, the paste was transferred into 24 moulds with the dimensions of 6 × 6 × 12 mm^3^ for each mixture. The samples were stored at 37 °C and 100 % humidity. After 1 d, 12 samples per mixture were removed from the storage and demoulded to be ready for the measurement. The second batch of 12 samples was handled the same, but after 7 d of storage. The measurement was performed at the universal testing machine Z010 (Zwick, Germany) with a crosshead speed of 1 mm/min until a major failure was produced.

#### 2.4.3. Porosity

The remaining parts of the samples used for the compressive strength tests after 1 d and 7 d of reaction time were used to measure the influence of the various concentrations of added sodium phytate on the porosity. For every measurement, a sample piece was put into a dilatometer and measured in the mercury porosimeters Pascal 140 and 440 (Thermo, Italy). At the Pascal 140, the dilatometer got evacuated to 0.01 kPa and filled with mercury. During the measurement at the Pascal 140, the pressure was increased linearly from 0.01 to 400 kPa. After that, the sample was transferred into the Pascal 440, where the pressure was increased from 0.1 to 400 MPa.

The size of the pores corresponding to each pressure was calculated by the Washburn Equation (2), where r is the calculated pore radius, γ the surface tension of mercury (480 dyne/cm), θ the contact angle of mercury (140°) and P the pressure in kPa:

(2) $$r=\frac{2\gamma *\cos\theta }{P}$$

With 1 kPa = 104 dyne/cm^2^, the equation can be simplified to:

(3) $$r=\frac{735403}{P}$$

Together with the volume change registered at the specific pressure, the relative pore volume can be calculated.

#### 2.4.4. SEM Visualization 

The pieces left from the compressive strength tests were used for SEM. These were coated with a 4 nm platinum layer in an ACE600 (Leica, Wetzlar, Germany) sputter coating unit and then put into a Crossbeam 340 (Zeiss, Oberkochen, Germany) SEM for the measurement. Secondary electron pictures were taken in a high vacuum, with an acceleration voltage of 2 kV.

### 2.5. Statistical Methods

For all of the quantitative XRD data, pH values, calorimetry data, BET and injectability measurements, the errors were determined as the standard deviations of three independently performed measurements. For the laser diffraction, three independent preparations with 10 measurement runs for each were analysed, and the 30 resulting data sets were averaged. Three independent preparations for the zeta potential measurement resulted in nine values that were used to determine the mean and the standard deviation. Not all prepared samples were suitable to use for the mechanical tests, but there was a minimum of nine independently prepared samples per mixture.

## 3. Results

### 3.1. Characterization of Powder Samples

#### 3.1.1. Particle Size Distribution and Specific Surface Area 

**Figure 1:**
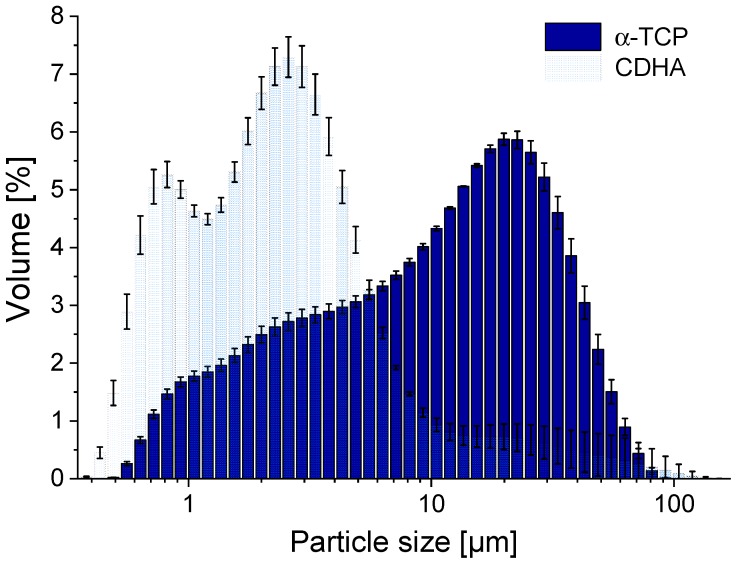
Particle size distribution of α–TCP and CDHA, determined by laser diffraction using isopropyl alcohol as a lubricant; the means of three independently prepared measurements are presented; the error bars represent the standard deviation.

The CDHA powder showed a bimodal particle size distribution, with maxima at particle sizes of 0.8 and 2.6 µm. The particle size distribution of α–TCP was unimodal with a maximum at 20 µm, while grain sizes reached down to 0.3 µm. Figure 1 and the D_v_ values (Table 1) indicate that the CDHA powder was basically finer than the α–TCP powder. 

#### 3.1.2. Quantitative Phase Composition 

The α–TCP starting powder was composed of 86 ± 1 wt.% crystalline α–TCP, 2.9 ± 0.1 wt.% β–TCP and 11 ± 1 wt.% amorphous TCP (ATCP). The G-factor quantification of the fine milled CDHA powder resulted in a crystalline CDHA content of 98.5 ± 0.7 wt.%, which means that practically no amorphization of CDHA had occurred during the wet milling procedure. For the model ellipsoid used to refine the anisotropic crystallinity of CDHA, values of rx = 5.73 ± 0.04 nm and rz = 16.5 ± 0.8 nm were obtained, which corresponds to a "true crystallite size" (True CS) of 13.1 ± 0.3 nm. A microstrain of 0.28 ± 0.02 was obtained. 

#### 3.1.3. Zeta Potential

**Figure 2:**
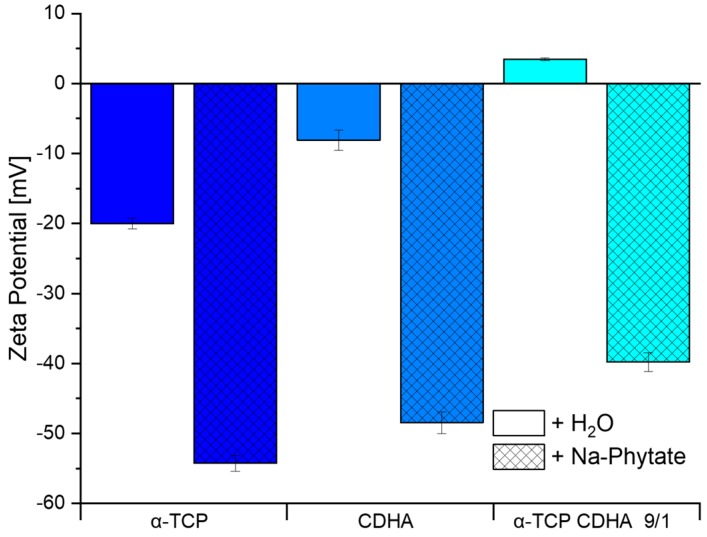
Surface charge of α-TCP, CDHA and a α-TCP/ CDHA mixture in water and a 0.002 M sodium phytate solution determined by the zeta potential measurement. Every powder was prepared independently three times, and the error bars represent the standard deviation.

The surface charge of these powders in deionized water and a sodium phytate solution is shown in Figure 2. It can be seen that the surface charge is much less negative in pure water or even slightly positive for the 9:1 powder mixture, compared to the sodium phytate solution. With the addition of sodium phytate in the solution, the surface charge decreased vastly by about 35 to 40 mV from −20 ± 1 mV to −55 ± 1 mV, −8 ± 1 mV to −48 ± 1 mV and 3 ± 0 mV to −40 ± 1 mV for α–TCP, CDHA and the mixture, respectively. 

### 3.2. Cement Paste Characterization 

#### 3.2.1. Viscosity

**Figure 3:**
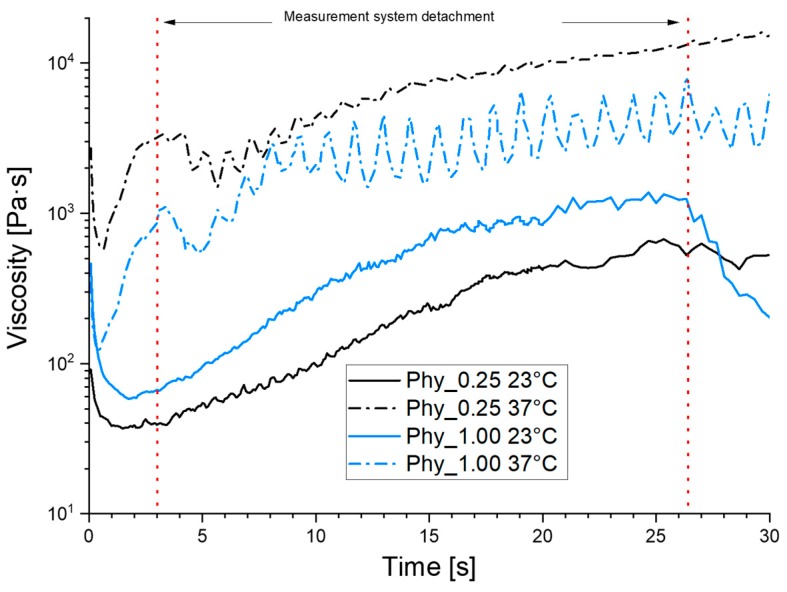
The paste viscosity development of the samples Phy_0.25 (black) and Phy_1.00 (blue) at 23 °C (line) and 37 °C (dotted line). The reference Phy_0.00 was not measurable. Every sample was prepared three times independently, and one representative curve is shown.

As shown in Figure 3, the pastes show a clear shear thinning behaviour in the early stages of the reaction. The reference Phy_0.00 was not measurable, as the paste was already too thick to be prepared for the rheometer. It can be seen that the added phytate has no significant increasing liquefying effect with an increasing amount in the paste. In contrast to this, a temperature increase from 23 °C to 37 °C has a strong effect. This is shown by the fast viscosity increase after the initial shear thinning period for the samples at 37 °C, compared to the slower increase for the 23 °C samples. When the sample surface is partially hardened, the measurement system can detach from the sample, which leads initially to a decrease in the measured viscosity until it attaches again.

#### 3.2.2. Injectability

**Figure 4:**
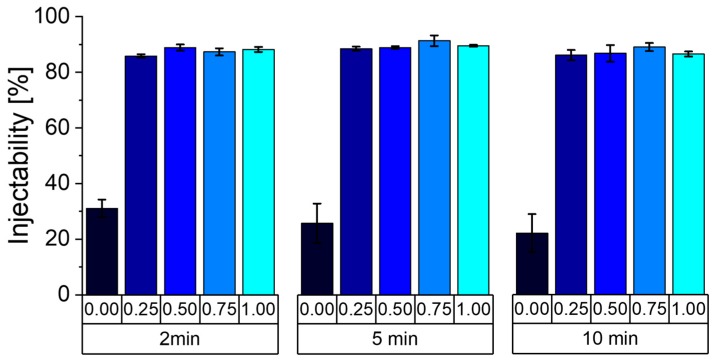
Injectability of all five paste formulations (Phy_0.00 – Phy_1.00) 2, 5 and 10 min after the powder and liquid mixing. Every sample has been prepared three times, and the error bars represent the standard deviation.

The reference paste Phy_0.00, without additional modification, showed a poor injectability after all three time points (Figure 4). The amount injected is mostly due to filter pressing. With 30% and less it is at least three times lower than that of all of the IP6 containing mixtures. All of them are shown to be fully injectable, as the last 10% that are missing stay in the needle when the syringe plunger reaches the end of the syringe. The injectability also does not change if the pastes can set for a longer time. These results show a significant improvement in the paste injectability, even with low amounts of 0.25 wt.% additional sodium phytate. 

### 3.3. Hydration Reaction

#### 3.3.1. Heat Flow Calorimetry

**Figure 5:**
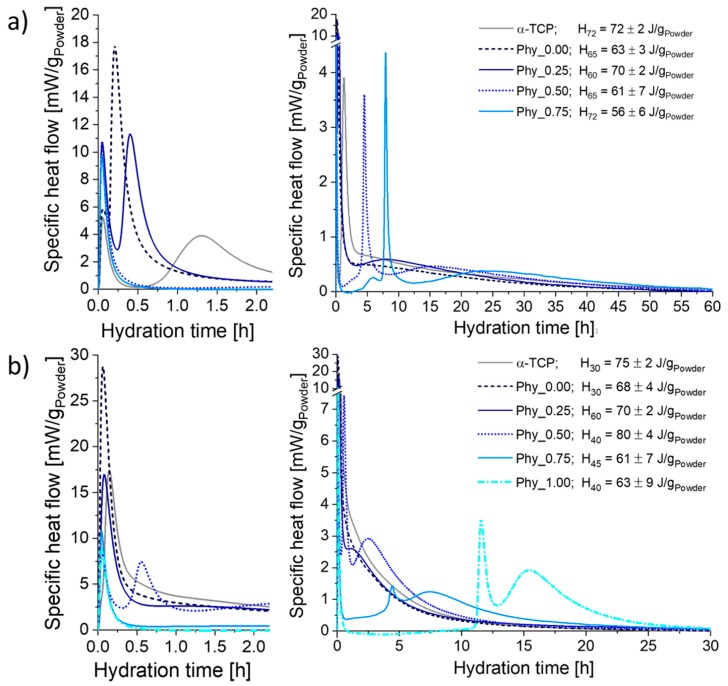
The calorimetry results of the samples composed of α–TCP and CDHA (weight ratio 9:1) with different amounts of sodium phytate; a 0.2 M Na_2_HPO_4_ solution was used as the mixing liquid with an L/P of 0.3 ml/g_Powder_. The measurements were performed at (**a**) T = 23 °C and (**b**) T = 37 °C; three independent measurements were run for each sample; one representative curve is shown for Phy_0.75 at T = 23 °C and for Phy_0.50, Phy_0.75 and Phy_1.00 at 37 °C, and for the other samples the mean of three independent measurements is presented.

The calorimetry curves obtained at 23 °C (Figure 5a) mainly exhibited three maxima: The initial heat flow already measured after a few min was followed by a sharp second maximum. Then, a third maximum occurred, which was flat and broad for all samples. While the position of the initial maximum was practically identical for all cement compositions, the maximum initial heat flow was higher for the samples containing phytate. The positions of both the second and the third maxima were shifted to later time points with an increasing phytate content. While the height of the second maximum decreased with a rising phytate content for Phy_0.00, Phy_0.25 and Phy_0.50, it increased again for Phy_0.75. The maxima of Phy_0.00 and Phy_0.25 were also sharper than those with higher phytate concentrations. The second maximum of pure α–TCP occurred later than that of the CDHA containing reference Phy_0.00, and the maximum heat flow that was reached was also far lower. For all of the samples, the measurements were well reproducible. 

The total heat release measured until the completion of hydration varied in the range of 56 ± 6 J/g_Powder_ (Phy_0.75) to 70 ± 2 J/g_Powder_ (Phy_0.25) for the samples containing CDHA. No systematic correlation between the phytate content and total heat release was observed. 

At 37 °C, only one heat flow maximum was visible for the samples without a phytate addition (Figure 5b). Sample Phy_0.25 showed two maxima: A small shoulder followed the sharp initial maximum. The samples with a higher phytate content exhibited three maxima, including the initial one. Here, the position of the second and third maximum was increasingly postponed with an increasing phytate content. In Phy_1.00, the heat flow completely decreased to zero and even reached slightly negative values after the initial maximum. This induction period lasted about 10 h. The measurements with IP6 concentrations up to 0.5 wt.% were well reproducible, while the reproducibility was lower for higher IP6 concentrations. For example, for Phy_1.00, the position of the second maximum varied from 9.6 to 11.5 h. As for 23 °C, no systematic correlation between the total heat release and the phytate content was observed. 

#### 3.3.2. pH Development

**Figure 6:**
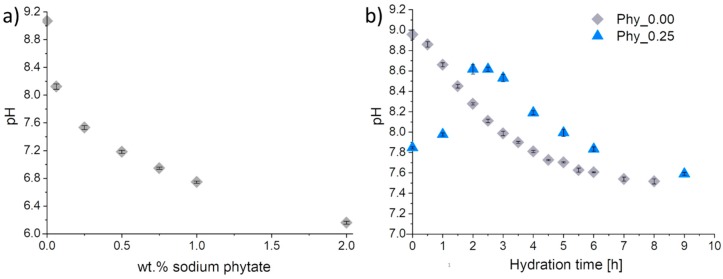
The pH measurements of (**a**) 0.2 M Na_2_HPO_4_ aqueous solutions containing variable amounts of sodium phytate (wt.% related to the powder content of the corresponding cement pastes) and (**b**) cements composed of α–TCP and CDHA (weight ratio 9:1) with 0 and 0.25 wt.% sodium phytate; a 0.2 M Na_2_HPO_4_ aqueous solution was used as the mixing liquid with an L/P of 1.2 ml/g_Powder_. The measurements were performed at T = 37 °C; three independent measurements were run for each sample, and the error bars represent the standard deviation.

While the 0.2 M Na_2_HPO_4_ solution was slightly basic with a pH of 9.07, the pH decreased with an increasing sodium phytate addition (Figure 6a), until it reached a value of 6.16 ± 0.02 for an addition of 2 wt.% sodium phytate. In Phy_0.00, a continuous decrease of the pH was observed immediately from the beginning (Figure 6b). After 8 h, a pH of 7.52 ± 0.03 was measured, while the initial pH (8.96 ± 0.05) was very close to that of the pure 0.2 M Na_2_HPO_4_ mixing liquid (9.07). In Phy_0.25, the starting pH was lower with 7.85 ± 0.01, but close to that of the mixing liquid containing the corresponding amount of sodium phytate (8.12 ± 0.04). Contrary to Phy_0.00, the pH of the cement paste in Phy_0.25 increased until 2 h after mixing, reaching a small plateau at a pH of 8.62 ± 0.05. After about 2.5 h, the pH started to decrease, similar to Phy_0.00. 

#### 3.3.3. In-situ XRD

##### Measurements at 37 °C 

**Figure 7:**
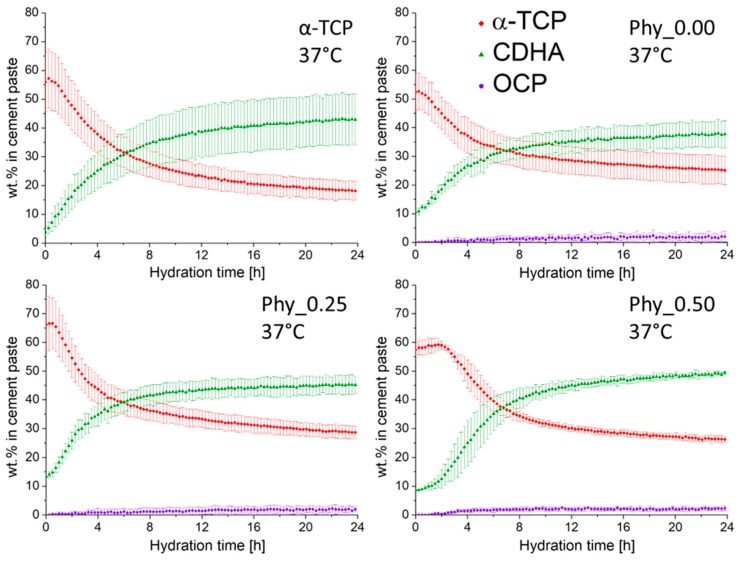
The quantitative in-situ XRD results of samples composed of α–TCP and CDHA (weight ratio 9:1) with different amounts of sodium phytate; a 0.2 M Na_2_HPO_4_ aqueous solution was used as the mixing liquid with an L/P of 0.3 ml/g_Powder_. The measurements were performed at T = 37 °C; three independent measurements were run for each sample, and the error bars represent the standard deviation; the data were corrected for the water loss occurring during the measurement.

The dissolution of crystalline α–TCP already started after about 0.25 h in α–TCP and Phy_0.00, while it was delayed to 0.5 h for Phy_0.25 and to 2 h for Phy_0.5 (Figure 7). An artefact of the initial increase of the α–TCP content was observed for all samples, while it was more pronounced for higher IP6 contents. This effect most likely results from the disappearance of a water film that might have been present at the beginning of the measurement, especially since the pastes with sodium phytate were rather liquid after preparation. A rapid increase of the CDHA content was observed since the beginning in α–TCP, Phy_0.00 and Phy_0.25, whereas it only started after 0.5 h in Phy_0.50. The increase became more rapid after about 2 h. A slight CDHA formation and crystalline α–TCP dissolution was measured until the end of the measurement time after 24 h for all samples that were investigated. The formation of minor amounts of OCP was detected in all samples, except α–TCP. 

##### Measurements at 23 °C 

**Figure 8:**
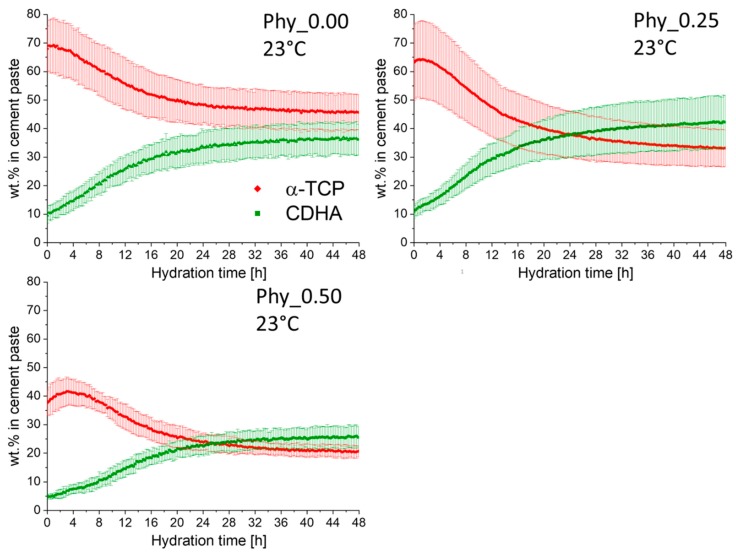
The quantitative in-situ XRD results of samples composed of α–TCP and CDHA (weight ratio 9:1) with different amounts of sodium phytate; a 0.2 M Na_2_HPO_4_ aqueous solution was used as the mixing liquid with an L/P of 0.3 ml/g_Powder_. The measurements were performed at T = 23 °C; three independent measurements were run for each sample, and the error bars represent the standard deviation.

At 23 °C, no OCP formation was detected in all of the samples investigated during the 48 h of measurement. Only the transformation of α–TCP to CDHA occurred, while the degree of hydration reached after 48 h was higher for both samples containing phytate (Figure 8). It further became evident that the dissolution of α–TCP was slower in these samples, compared to Phy_0.00. While the hydration reaction slowed down noticeably after about 25 h in all cases, the reaction still proceeded to a slight extent until the completion of the measurement in the phytate containing samples. In Phy_0.00, the contents of both α–TCP and CDHA reached a constant level at the end. 

### 3.4. Characterization of Hydration Products

#### 3.4.1. XRD of Hardened, Polished Samples 

##### Samples stored at 37 °C 

After 1 d of hydration, OCP was detected in small amounts in all samples except pure α–TCP (Table 2). The CDHA content after 1 d was identical for α–TCP, Phy_0.00 and Phy_0.50 within the error range, while it was significantly lower for Phy_1.00. Accordingly, the amount of remaining α–TCP was significantly higher for Phy_1.00. After 7 d, a further increase of CDHA quantities compared to 1 d was observed for all samples, though the difference was not significant for Phy_1.00. While no significant differences were observed between α–TCP, Phy_0.00 and Phy_0.50 for both α–TCP and CDHA, the CDHA content was still far lower for Phy_1.00, compared to the other samples. As for 1 d, OCP was detected in all samples except α–TCP, while the quantity increased in the range of Phy_0.50, Phy_0.00/Phy_2.00 and Phy_1.00.

The degree of hydration (defined as the percentage of TCP phases (ATCP + α–TCP) that have reacted) in the samples stored for 1 d, was compared to that reached in the in-situ XRD measurements after 1 d of hydration. It was evident that the hydration proceeded to a higher extent in the storage samples for Phy_0.00 and slightly for Phy_0.50, while no significant difference was observed for α–TCP. 

A further increase of the crystallite size True CS was only observed for Phy_0.50 from 1 d to 7 d (Table 3). The aspect ratio rz/rx was practically identical for all samples expect α–TCP, and slightly higher than that in the CDHA added as starting powder. 

##### Samples stored at 23 °C 

For all of the samples investigated, a continuous decrease of the α–TCP content was observed up to 7 d (Table 4). While in the samples stored for 1 d or 2 d the α–TCP content was still significantly higher and the CDHA content was lower in Phy_1.00, compared to the other two samples, nearly no differences were observed anymore after 4 d and 7 d. No crystalline OCP was detected in all of the samples that were hydrated for 1 d and 2 d. Indeed, the main XRD reflection of OCP was clearly visible in the diffraction patterns of the samples stored for 4 d and 7 d. Accordingly, OCP could be quantified in all of these samples. The degree of hydration in the samples stored at 23 °C continuously increased up to a hydration time of 7 d (Table 5). While it was lower for Phy_1.00, compared to the other two samples, after 1 d and 2 d, similar values were obtained for all three samples after 4 d and 7 d. 

A slight increase of the crystallite size True CS from 1 d up to 4 d of hydration was observed for Phy_0.00 and Phy_0.50 (Table 6). For Phy_1.00, a decrease of True CS was measured from 1 d to 2 d, but the differences were within the error range. After 7 d, only marginal differences in True CS could be detected between the different samples. The aspect ratio rz/rx was also comparable for all samples after 7 d. While the values obtained after 1 d differed from the others, after 2 d a constant level was reached.

#### 3.4.2. Compressive Strength

**Figure 9:**
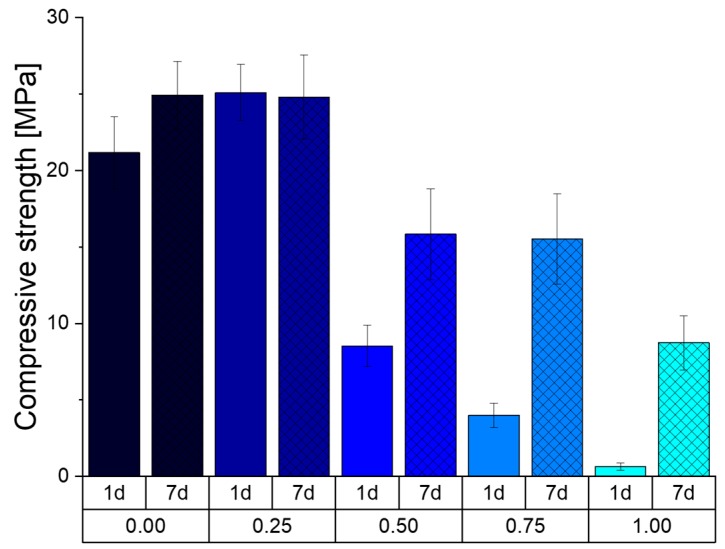
The compressive strength of all five paste formulations (0.00–1.00) after 1 d and 7 d at 37 °C and 100% humidity. Every sample has been prepared independently at least 9 times, and the error bars represent the standard deviation.

Figure 9 shows the compressive strength (CS) of the prepared cement cuboids after storage at 37 °C and 100% humidity. It can be seen that the formulation Phy_0.00, without the addition of sodium phytate, initially has a lower CS of 21 MPa compared to the formulation Phy_0.25 with 25 MPa. All other formulations show a significant lower CS with 8, 4 and 0.5 MPa for the samples Phy_0.50, Phy_0.75 and Phy_1.00, respectively. After 6 more days of storage, the CS of the samples Phy_0.25 remained at about 25 MPa, and the CS of Phy_0.00 rose to the same CS. The CS for Phy_0.50, Phy_0.75 and Phy_1.00 also increased to the respective values 16, 15.5 and 9 MPa.

#### 3.4.3. Porosity 

**Figure 10:**
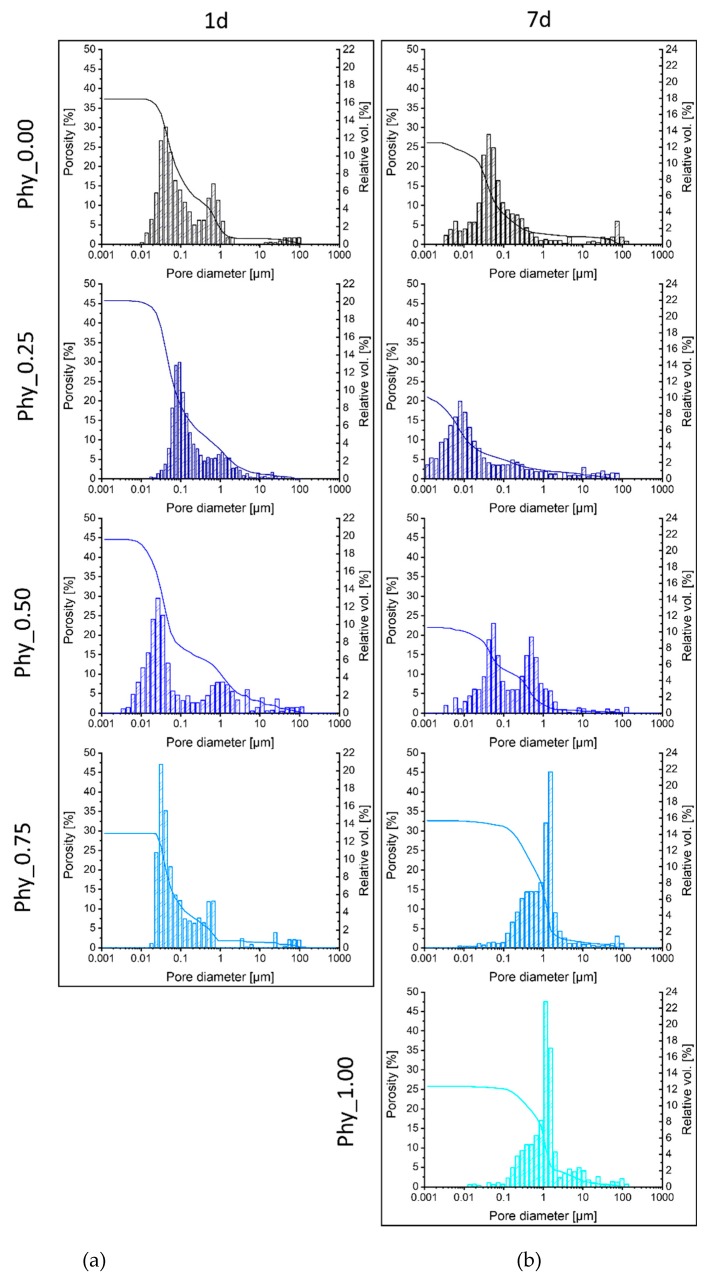
The pore diameter distribution and total porosity of all mixtures after (**a**) 1 d and (**b**) 7 d of hydration at 37 °C and 100% humidity. The sample Phy_1.00 was not hardened after 1 d, so it was not possible to measure the pores.

The porosimetry data presented in Figure 10 (a for 1d and b for 7d) and Table 7 show the effects of the sodium phytate addition as well as the effect of the storage time on the development of pores of different sizes and volume. The overall total pore volume accessible by Hg porosimetry decreased during the storage of the samples Phy_0.00, Phy_0.25 and Phy_0.50 from 37% to 26%, 46% to 21% and 45% to 22%, respectively. The total porosity for the sample Phy_0.75 remains roughly the same over the 7 d of storage. The sample Phy_0.75 shows an increase in the total pore volume, and Phy_1.00 is not hardened after 1 d. The shrinkage of the pores can be best seen in the sample Phy_0.00, where the average diameter halves from 0.06 µm to 0.03 µm, but this is also present in other samples. With the addition of sodium phytate, the overall pore size after 1 d gets decreased at first from a bimodal distribution with two maxima at 0.03 µm and 0.1 µm for Phy_0.00 to a nearly monomodal distribution with a maximum at 0.1 µm and a small shoulder at 1 µm for the sample Phy_0.25. Nearly the same distribution is seen in the sample Phy_0.50, which is very similar to Phy_0.00 regarding the bimodal distribution. Phy_0.75 shows a higher number of smaller pores, which have their maximum at 0.28 µm. The total pore volume seems to be independent of the amount of added sodium phytate, as it is mostly regulated by the liquid content, which is equal throughout all of the samples.

#### 3.4.4. SEM Images 

**Figure 11:**
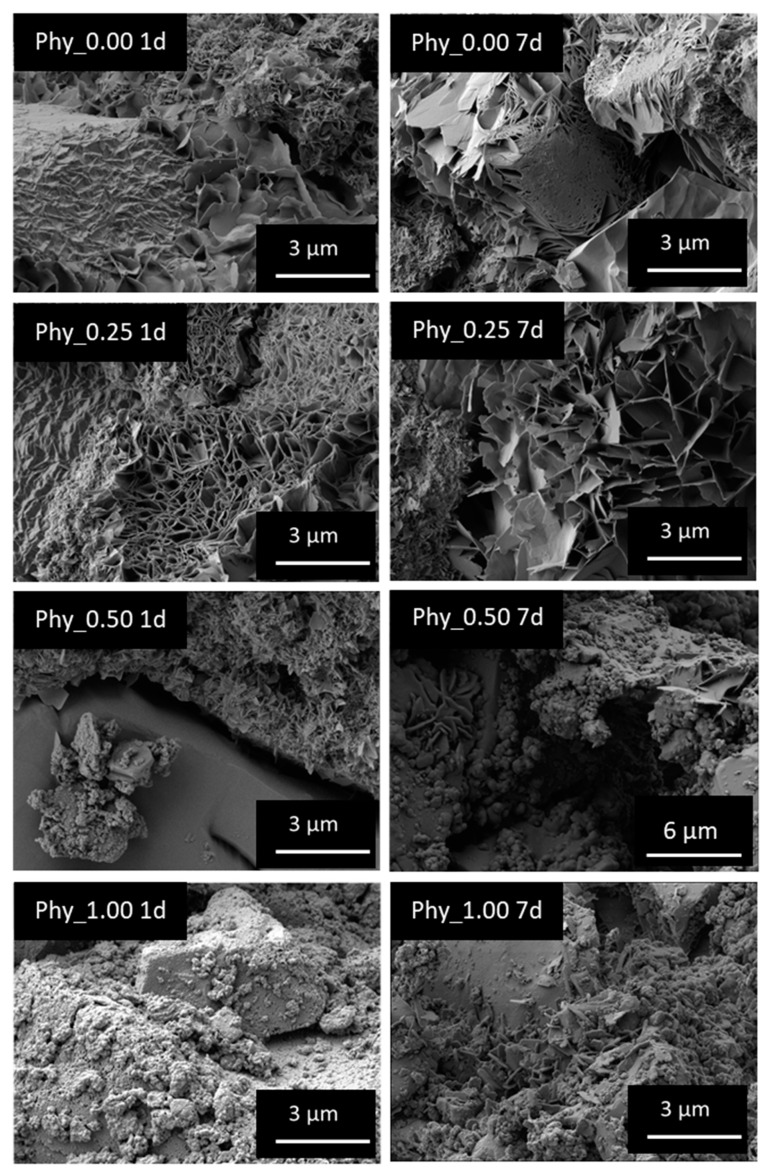
CDHA crystals of the mixtures Phy_0.00, Phy_0.25, Phy_0.50 and Phy_1.00 grown for 1 d and 7 d at 37 °C and 100% humidity, visualized with the SEM. All samples have been observed on different areas, and representative images were selected for presentation.

The delayed precipitation reaction can also be observed via the SEM. In Figure 11 the crystals for Phy_0.00 and the mixtures Phy_0.25, Phy_0.50 and Phy_1.00 are shown side by side after 1 d and 7 d of hydration. After 1 d, it can clearly be seen that the sample with the least amount of phytate shows no apparent difference to Phy_0.00. Both samples show plate-like crystals with a size of just under 3 µm. The mixture Phy_0.50 shows very small crystals, and Phy_1.00 does not show any distinguishable crystals at all. After 7 d, the crystals in Phy_0.00 and Phy_0.25 have grown larger and have become heavily entangled. Now, in Phy_0.50 and Phy_1.00 crystals with sizes of up to 1 µm can also be seen.

## 4. Discussion

### 4.1. Effect of CDHA Filler on the Hydration

A comparison of the hydration of Phy_0.00 (weight ratio α-TCP/CDHA = 9:1) with the pure α–TCP powder demonstrated that the CDHA had an accelerating effect on the hydration. This was clearly visible in the calorimetry measurements performed at 23 °C and it was slightly visible at 37 °C. The fine CDHA powder most likely acts as a nucleation site during the cement hydration [7]. 

### 4.2. Effect of Sodium Phytate on the Setting Mechanism of the Cement

The calorimetry measurements indicated that all samples, including those containing sodium phytate, showed the typical reaction mechanism established for α–TCP containing ATCP [24]. 11 ± 1 wt.% of ATCP were present in the α–TCP starting powder used in this study. The hydration of ATCP, indicated by a sharp maximum in the calorimetry measurements, is followed by a slower reaction of the crystalline α–TCP. This is supported by the in-situ XRD data, where the CDHA formation started earlier than the dissolution of crystalline α–TCP. In the not retarded mixtures α–TCP and Phy_0.00, ATCP hydration most likely proceeded so fast that this reaction cannot be separated from the initial heat flow resulting from the mixing of the cement paste. The shift of the second, small calorimetry maximum observed for the phytate-containing samples corresponds well to the retarded α–TCP dissolution measured by the in-situ XRD.

Sodium phytate was proven to have a strong retarding effect on the hydration of α–TCP, even in small concentrations below 1 wt.%. Higher concentrations (Phy_0.75 at 23 °C and Phy_1.00 at 37 °C) even resulted in a real induction period. This means that the hydration of the highly reactive ATCP was also fully impeded during an induction period of about 10 h in Phy_1.00. As expected, the reaction was generally accelerated by increasing the temperature from 23 °C to 37 °C for all cement compositions, while the general reaction kinetics remained the same. 

In order to further clarify the hydration mechanism, the pH measurements were performed and correlated to the in-situ XRD measurements. The continuous decrease of the pH observed for Phy_0.00 could be clearly related to the in-situ XRD measurement performed at 37 °C, where the α–TCP dissolution and CDHA formation were observed from the beginning (Figure 7). The pH resulting from the dissolution of α–TCP in deionized water was determined to be around 9.2 at 37.4 °C [22] and 9.6 [32] in other studies, respectively; hence, α–TCP shows an alkaline reaction. During the formation of CDHA, the ions released from the dissolution of ATCP and α–TCP are consumed according to Equation (5), taking into account that the H_2_PO_4_^-^ and HPO_4_^2−^ species are present at the pH conditions in the cement pastes that were investigated, while the ratio of these species depends on the exact pH [33].

9 Ca^2+^ + *x* HPO_4_^2-^ + (6 − *x*) H_2_PO_4_^-^ + (12-*x*) OH^-^ → Ca_9_(PO_4_)_5_(HPO_4_)OH + (11 − x) H_2_O *(0 ≤ x ≤ 6)* (5)

Reaction (5) results in a decrease of the pH toward more neutral values due to the precipitation of CDHA, which involves the consumption of OH^−^ ions. This was observed in the current measurements and also reported from other investigations [20,21]. 

A clear difference was noticed for the phytate containing sample, where an initial increase of the pH was measured. Here, a neutralization reaction between the slightly acidic phytate and the alkaline ions resulting from the dissolution of ATCP and α–TCP might occur, with the result that the pH of the solution approaches that of the initial Na_2_HPO_4_ solution. Then, due to the precipitation of CDHA, the equilibrium pH is reached, as in the reference. After the completion of the reaction, the pH is close to neutral in both cases. 

As the pH of the mixing liquid decreased with an increasing sodium phytate concentration, this factor might have affected the speed of the hydration reaction, since the ratio of the H_2_PO_4_^−^ and HPO_4_^2−^ species is affected by the pH. Actually, the pH of the mixing liquid was reported to mainly have an effect on the initial part of the hydration reaction, which occurred earlier for lower pH values [7]. Contrary to this, in the present study a decrease in the pH due to the phytate addition resulted in a retardation of the setting reaction. Therefore, it is unlikely that the pH is the relevant factor explaining the setting retardation. Another explanation might be a decrease of the availability of free Ca^2+^ ions through the formation of chelate complexes with the phytate [34], which would impede the precipitation of CDHA. Thus, the retarding effect can be compared to that of phytic acid in brushite cement [18,19]. 

The storage samples hydrated at 37 °C did not show a systematic relation between the phytate content and the amount of OCP formed in the sample. Sample Phy_2.00, which should have shown the highest OCP formation, was well within the content of the other samples. These observations suggest that the OCP formation in relation to CDHA is not significantly affected by the phytate addition and the variation of the phytate content. Interestingly, at 23 °C, OCP was only detected after 4 d and 7 d for all of the investigated samples (Phy_0.00. Phy_0.50 and Phy_1.00). Therefore, it can be concluded that the OCP formation started later than the CDHA formation at a lower temperature of 23 °C. 

OCP, whose crystal structure has similarities to that of hydroxyapatite [35], is frequently reported as a transient intermediate in the formation of the more stable apatite from solution [36]. In the present study, no decrease of the OCP content was observed in the in-situ XRD measurements during the respective measurement time. Additionally, as mentioned earlier, the formation of OCP occurred later than the CDHA formation at 23 °C, which means that OCP does not act as a typical precursor in these cases, but rather forms as a by-product during the CDHA formation. Still, it should be kept in mind here that XRD is only sensitive to crystalline phases and hence it might be possible that an amorphous precursor of OCP is already formed earlier, as is also reported in the literature [37].

As only a few wt.% of OCP were present in the samples that were here investigated, it is expected not to have any pronounced effect on the biological performance of the hardened cements. If any effect at all would be present, it is expected to be a positive one, as OCP is reported to have a promoting effect on bone formation [38]. 

The quantitative XRD investigations of the storage samples demonstrated that at 23 °C, the quantitative phase composition obtained after 7 d was similar for all phytate concentrations that were investigated, while significant differences were obtained for the samples stored at 37 °C. Therefore, it appears that at 37 °C, the phytate reduces the overall extent of the reaction when a content of 0.5 wt.% is exceeded. Interestingly, this is not the case at 23 °C. 

The crystallinity (size of coherent scattering domains) of the CDHA formed after hydration was not noticeably affected by the addition of sodium phytate. In other studies, the crystallite sizes of CDHA resulting from α–TCP hydration were remarkably affected by certain parameters, like the particle size [39] or amorphous content of the starting powder [24,40]. Here, the authors explained this by the acceleration of the hydration by either the reduction of the particle size or an increasing amorphous content. A faster reaction leads to a higher degree of supersaturation of the solution, which favours nuclei formation and hence the precipitation of smaller crystals [40]. Apparently, this was not the case for the phytate-containing samples investigated in this study – otherwise, the phytate containing samples would have achieved higher CDHA crystallinities. Instead, at 37 °C, phytate concentrations of 1 and 2 wt.% slightly reduced the crystallite sizes, which correlates with the reduced degree of hydration observed in these samples. Hence, there are indications that phytate in higher concentrations has an impeding effect on CDHA crystallite growth.

The SEM images after 1 d and 7 d reveal a clear reduction of the CDHA crystal size for phytate concentrations exceeding 0.25 wt. Hence, the growth of CDHA crystals is impeded by the phytate, while the crystallites detected by XRD are only affected to a minor extend. As the crystal size is in the range of a few µm, while the crystallites are only between 10 and 20 nm, it is obvious that the crystals visible under SEM are mosaic crystals composed of several crystallites [41].

### 4.3. Effect of Sodium Phytate on the Processability

The addition of sodium phytate resulted in a more negative zeta potential. A similar observation was made for brushite cement modified with phytic acid in a previous study [19]. This is well in accordance with the earlier studies of Takahashi et al. [34]. The phytate has six phosphate groups, which result in a strong negative surface charge, when the phytate reacts with the free calcium near the cement particle surface. This change in the overall surface charge of the powder is believed to enhance the particle dispersion and inhibit aggregate formation due to the mutual repulsion of the particles [34]. In a previous study [19], the amount of sodium phytate could be correlated with the initial paste viscosity, but the measurements here do not show this trend. This might be due to the fact that the system is already saturated with only 0.25 wt.% sodium phytate, which is also backed by the injectability tests. Here, a significant improvement in injectability can be seen with the addition of 0.25 wt.% sodium phytate, but no further improvement result from higher amounts, even at later time points of 5 and 10 min after mixing. Nevertheless, just like in the previous study, the paste stays injectable for significantly longer with sodium phytate. This can be explained by the chelate complex formation between Ca^2+^ ions and the phosphate groups of the IP6, which are likely less soluble than α–TCP. In addition, the complex formation additionally slows down the solution/precipitation cement reaction by decreasing the availability of free calcium ions [42,43,44]. This leads to a delayed rise in paste viscosity and also provides a good explanation for the strong retarding effect on cement hydration, as was clearly demonstrated in this study. The most impactful factor controlling the paste viscosity seems to be the reaction temperature. With an increase from 23 °C to 37 °C, the early viscosity increase is hugely accelerated. Again, there is no noticeable difference with a higher amount of sodium phytate.

### 4.4. Effect of Sodium Phytate on the Mechanical Properties

The delay in the reaction is also visible in the compressive strength measurements. Here, a sodium phytate content higher than 0.25 wt.% resulted in very low compressive strengths after 1 d and only slightly higher ones after 7 d. This is due to the fact that the reaction progresses less with more sodium phytate. The samples Phy_0.00 – Phy_0.75 show about the same initial porosity of approx. 40 %. This can be explained by the fact that the porosity is mostly regulated by the amount of liquid added to the system, and this is kept constant throughout all samples. The remarkable decrease of the total pore volume measured for samples containing up to 0.5 wt.% phytate appears surprising. As the densities of α–TCP and CDHA (2.86 g/cm^3^ and 3.13 g/cm^3^, respectively) are very similar, the total volume of the solid content is not expected to increase during hydration. The total pore volume might be reduced due to the shrinkage of the samples as a result of drying. Still, since the samples were stored at 100 % humidity, this effect can be excluded in this study. As a shift of the pore size distribution toward smaller diameters was observed, it is likely that the formation of CDHA crystals inside the pores led to the separation of pores into smaller ones. During this process, parts of the pore space might have been closed. These closed pores are not accessible by Hg porosity measurements, which would explain the apparent reduction of the total pore volume. Parts of the pores might also be so small that the limit of the measurement technique is reached. No direct correlation between the pore size and distribution, and the amount of added sodium phytate can be made. 

## 5. Conclusions

Sodium phytate was proven to be a versatile additive for the development of an apatite cement with excellent injectability. An amount of 0.25 wt.% was already sufficient to achieve maximum injectability. The increase in injectability could be related to a decrease in the particles´ zeta potential. While a phytate amount of 1 wt.% had a drastic retarding effect on cement hydration, 0.25 wt.% of phytate only slightly affected the setting kinetics. Furthermore, this low amount of the additive had nearly no influence on the final phase composition, compressive strength, the XRD crystallinity and the crystal size of the CDHA after hardening. Therefore, a sodium phytate concentration of 0.25 wt.% can be considered as optimum for achieving a CDHA-forming cement with an optimum injectability without negatively affecting other properties at the physiological temperature of 37 °C. Higher concentrations are not recommended due to the intense retarding effect on cement setting.

## Figures and Tables

**Table 1:** D_V_ values obtained by laser diffraction and BET surface areas of α–TCP and CDHA; the values are the means of three independent preparations, while for the laser diffraction ten measurement runs were performed for each preparation; the errors represent the standard deviations of the three preparations.

Parameter	α-TCP	CDHA
D_V_10 [µm]	1.6 ± 0.1	0.73 ± 0.02
D_V_50 [µm]	11.3 ± 0.8	2.3 ± 0.1
D_V_90 [µm]	35 ± 1	10 ± 5
BET surface area [m^2^/g]	0.86 ± 0.06	34 ± 4

**Table 2:** The quantitative phase composition and degree of hydration of samples composed of α–TCP and CDHA (weight ratio 9:1) with different amounts of sodium phytate after 1 d and 7 d of hydration; a 0.2 M Na_2_HPO_4_ aqueous solution was used as the mixing liquid with an L/P of 0.3 ml/g_Powder_; the samples were stored at 37 °C.

	wt.% α–TCP		wt.% CDHA		wt.% OCP		Degree of Hydration [%]		
Hydration Time	1 d	7 d	1 d	7 d	1 d	7 d	1 d	7 d	In-situ 1 d
α–TCP	20 ± 1	9 ± 1	50 ± 2	61 ± 1	0	0	72 ± 1	87 ± 1	71 ± 1
Phy_0.00	16 ± 1	10 ± 2	47 ± 6	56 ± 4	2.5 ± 0.4	2.0 ± 0.3	72 ± 3	83 ± 3	56 ± 4
Phy_0.50	20 ± 2	6 ± 2	49 ± 2	60 ± 1	3.0 ± 1.1	0.5 ± 0.8	68 ± 3	90 ± 3	62 ± 1
Phy_1.00	33 ± 3	28 ± 9	31 ± 6	37 ± 9	1.9 ± 0.4	3.6 ± 0.4	44 ± 7	54 ± 14	-
Phy_2.00	-	17 ± 5	-	44 ± 7	-	2.2 ± 0.4	-	69 ± 10	-

**Table 3:** True CS and aspect ratio rz/rx of CDHA crystallites in storage samples after 1 d and 7 d of hydration; a 0.2 M Na_2_HPO_4_ solution was used as the mixing liquid with an L/P of 0.3 ml/g_Powder_; the samples were stored at 37 °C.

	True CS [nm]		Aspect Ratio rz/rx	
Hydration time	1 d	7 d	1 d	7 d
**CDHA powder**	13.1 ± 0.2	13.1 ± 0.2	2.9 ± 0.1	2.9 ± 0.1
**α–TCP**	14.1 ± 0.2	14.8 ± 0.7	5.3 ± 0.5	5.4 ± 0.4
**Phy_0.00**	13.8 ± 0.6	15.1 ± 0.8	3.6 ± 0.2	3.5 ± 0.4
**Phy_0.50**	11.4 ± 0.4	16.2 ± 0.8	3.6 ± 0.1	4.5 ± 0.4
**Phy_1.00**	10 ± 2	10.1 ± 0.2	3.4 ± 0.1	3.6 ± 0.1
**Phy_2.00**	-	9.5 ± 0.6	-	3.9 ± 0.1

**Table 4:** The quantitative phase composition of samples composed of α–TCP and CDHA (weight ratio 9:1) with different amounts of sodium phytate after 1 d, 2 d, 4 d and 7 d of hydration; a 0.2 M Na_2_HPO_4_ aqueous solution was used as the mixing liquid with an L/P of 0.3 ml/g_Powder_; the samples were stored at 23 °C.

	wt.% α–TCP				wt.% CDHA				wt.% OCP			
Hydration Time	1 d	2 d	4 d	7 d	1 d	2 d	4 d	7 d	1 d	2 d	4 d	7 d
**Phy_0.00**	34 ± 4	30 ± 1	23 ± 1	16 ± 1	36 ± 1	63 ± 1	64 ± 2	58 ± 4	0	0	4.1 ± 0.2	2.0 ± 0.2
**Phy_0.50**	39 ± 3	32 ± 2	22 ± 1	13 ± 2	36 ± 2	64 ± 5	61 ± 3	57 ± 7	0	0	3.7 ± 0.4	1.8 ± 0.6
**Phy_1.00**	58 ± 4	41 ± 3	21 ± 5	15 ± 3	15.0 ± 0.5	49 ± 4	56 ± 1	60 ± 2	0	0	4 ± 1	0.9 ± 0.1

**Table 5:** The degree of hydration of samples composed of α–TCP and CDHA (weight ratio 9:1) with different amounts of sodium phytate after 1 d, 2 d, 4 d and 7 d of hydration; a 0.2 M Na_2_HPO_4_ aqueous solution was used as the mixing liquid with an L/P of 0.3 ml/g_Powder_; the samples were stored at 23 °C.

	Storage Samples				In-situ	
Hydration Time	1 d	2 d	4 d	7 d	1 d	2 d
**Phy_0.00**	46 ± 3	64 ± 1	72 ± 1	76 ± 1	34 ± 3	38 ± 4
**Phy_0.50**	43 ± 4	63 ± 1	72 ± 1	80 ± 1	44 ± 1	51 ± 1
**Phy_1.00**	12 ± 2	49 ± 1	71 ± 5	78 ± 4	42 ± 2	50 ± 1

**Table 6:** True CS and aspect ratio rz/rx of CDHA crystallites in the storage samples after 1 d, 2 d, 4 d and 7 d of hydration; a 0.2 M Na_2_HPO_4_ solution was used as the mixing liquid with an L/P of 0.3 ml/g_Powder_; the samples were stored at 37 °C.

	True CS [nm]				Aspect Ratio rz/rx			
Hydration Time	1 d	2 d	4 d	7 d	1 d	2 d	4 d	7 d
**Phy_0.00**	10.4 ± 0.8	11.0 ± 0.4	12.0 ± 0.4	12.1 ± 0.1	4.2 ± 0.4	3.5 ± 0.2	3.4 ± 0.3	3.3 ± 0.3
**Phy_0.50**	9.0 ± 0.4	9.8 ± 0.3	11.0 ± 0.3	11.2 ± 0.2	4.5 ± 0.2	3.8 ± 0.1	3.9 ± 0.1	3.6 ± 0.2
**Phy_1.00**	9 ± 1	8.1 ± 0.3	10.7 ± 0.3	12 ± 1	2.7 ± 0.5	3.9 ± 0.1	3.9 ± 0.2	3.8 ± 0.3

**Table 7:** The total pore volume, porosity and average pore size of all samples stored for 1 d and 7 d at 37 °C and 100% humidity, measured by Hg-porosimetry.

Parameter	Storage time	Phy_0.00	Phy_0.25	Phy_0.50	Phy_075	Phy_1.00
Total pore volume [cm^3^/g]	1 d	0.17	0.21	0.19	0.12	-
	7 d	0.13	0.11	0.10	0.18	0.14
Total porosity [%]	1 d	37	46	45	30	-
	7 d	26	21	22	32	25
Average pore size [µm]	1 d	0.06	0.05	0.03	0.05	-
	7 d	0.03	0.04	0.05	0.04	0.05
